# Vaccine Cold Chain Performance Across Seven Provinces and Two Seasons in Nepal: Findings from a Nationwide Temperature Monitoring Study

**DOI:** 10.3390/vaccines14100837

**Published:** 2026-09-23

**Authors:** Abhiyan Gautam, Pawan Jung Rayamajhi, Bibek Kumar Lal, Madan Kumar Upadhyaya, Keshav Raj Pandit, Chetan Nidhi Wagle, Sanjeev Sharma Kattel, Bibek Shrestha, Swatantraraj Joshi, Krishna Prasad Subedi, Budhi Setiawan, Prakash Chandra Joshi, Nawaraj Khadka, Chandra Prakash Yadav, Mojtaba Haghgou, Hendrik Stapel, Achyut Bikram Thapa, Badebabu Thapa, Tilasha Bajracharya, Sajani Manandhar, Gambhir Shrestha, A.S.M. Shahabuddin, Adhish Dhungana, Khoja Fazil Ahmad Fazil

**Affiliations:** 1 Child Health Immunization Services, Family Welfare Division, Department of Health Services, Ministry of Health and Food Safety, Teku, Kathmandu 7830, Nepal; gautamabhiyan@gmail.com; 2 Curative Services Division, Department of Health Services, Ministry of Health and Food Safety, Teku, Kathmandu 7830, Nepal; pawanjungrayamajhi@gmail.com; 3 Department of Health Services, Ministry of Health and Food Safety, Teku, Kathmandu 7830, Nepal; bibeklal@outlook.com; 4 Family Welfare Division, Department of Health Services, Ministry of Health and Food Safety, Teku, Kathmandu 7830, Nepal; drmadannepal@gmail.com; 5 Management Division, Department of Health Services, Ministry of Health and Food Safety, Teku, Kathmandu 7830, Nepal; panditkeshavraj@gmail.com (K.R.P.); tilasha000@gmail.com (T.B.); 6 Logistics Management Section, Management Division, Department of Health Services, Ministry of Health and Food Safety, Teku, Kathmandu 7830, Nepal; uchetan@hotmail.com (C.N.W.); sanjeevsharma.kattel@nepal.gov.np (S.S.K.); swatantraraj@yahoo.com (S.J.); krishnasubedi@gmail.com (K.P.S.); 7 National Medical Laboratory, Department of Drug Administration, Ministry of Health and Food Safety, Kathmandu 10038, Nepal; bibekshrestha989@gmail.com; 8 United Nations Children’s Fund (UNICEF), Nepal Country Office, UN House, Pulchowk, Lalitpur, Kathmandu 1187, Nepal; bsetiawan@unicef.org (B.S.); pcjoshi@unicef.org (P.C.J.); nakhadka@unicef.org (N.K.); cyadav@unicef.org (C.P.Y.); adhungana@unicef.org (A.D.); 9 affectMD Inc., Toronto, ON M1R 4Z3, Canada; m.haghgou@affectmd.ca (M.H.); h.stapel@affectmd.ca (H.S.); a.abthapa@gmail.com (A.B.T.); phrbadebabu@gmail.com (B.T.); 10 New ERA, Rudramati Marga, Kalopul, Kathmandu 722, Nepal; sajani@newera.com.np; 11 Department of Community Medicine and Public Health, Maharajgunj Medical Campus, Institute of Medicine, Tribhuvan University, Kathmandu 1524, Nepal; gamvir.stha@gmail.com; 12 Maternal, Newborn, Child and Adolescent Health Section, Global Health Practice, Child Survival Centre of Excellence, United Nations Children’s Fund (UNICEF), Nairobi 67578-00200, Kenya; ashahabuddin@unicef.org

**Keywords:** vaccine cold chain, temperature monitoring, Nepal, mean kinetic temperature, freeze exposure, effective vaccine management, immunization supply chain

## Abstract

Background: Maintaining vaccines at 2–8 °C from manufacture to administration is a prerequisite for potency, yet cold chain failures remain common in low- and middle-income countries. The World Health Organization (WHO) recommends that every country conduct a temperature monitoring study (TMS) at least once every five years to identify and correct weaknesses in its vaccine supply chain. Nepal’s first national TMS was conducted in 2015–2016 and reported in 2018. The second was conducted seven years later, in 2025. We report its findings across both winter and summer seasons. Methods: Two cross-sectional temperature monitoring studies were conducted across seven supply chain routes, one per province, from the Central Vaccine Store (CVS) in Kathmandu to service delivery points. Study 1 (winter, January to March 2025) covered six routes; Study 2 (summer, April to July 2025) covered all seven. A total of 24 facilities across five supply chain levels were included, selected to represent Mountain, Hill, and Terai districts, rather than a statistically representative sample of all such facilities nationwide. WHO-prequalified data loggers (LogTag UTRID-16, primary; Vac-TL-01, backup) recorded temperatures at 30-min intervals. Two vaccines were tracked: Pentavalent (freeze-sensitive) and oral polio vaccine (OPV, heat-sensitive). Mean kinetic temperature (MKT) and percentage of time in the 2–8 °C range were the primary outcome measures. Results: Total monitoring time was 18,187 h (7787 winter; 10,400 summer). The mean percentage of time the vaccines were within the safe 2–8 °C range was 98.1% (95% CI 96.3–99.6%) of monitored time in winter (Study 1) and 97.4% (95% CI 95.4–99.1%) in summer (Study 2). However, vaccines were below 2 °C for a cumulative 272.5 h (1.5% of total time), concentrated in storage at the cold chain point and service delivery point levels, with one route recording −12.1 °C. Heat excursions above 8 °C totaled 155.0 h (0.9%), concentrated in storage at the central and provincial vaccine stores and in the transit between them. Vaccine vial monitors (VVM) were all at the usable stage, but slightly faded VVM readings for OPV indicated cumulative thermal stress. Conclusions: Nepal’s vaccine cold chain remained within 2–8 °C for more than 97% of monitored time in both seasons, and MKT values stayed within the recommended range; however, route-level data identified persistent sub −2 °C exposures (cumulatively 272.5 h, with one route reaching −12.1 °C). Because mean kinetic temperature is heat-weighted and the study did not include shake testing, the integrity of Pentavalent vaccine on routes with sub-zero excursions cannot be confirmed from these data alone. Recommendations requiring minimal capital investment, differentiated by season and route, include use of temperature-regulated loading areas, ice-pack conditioning training, updated standard operating procedures (SOPs) for transit, refrigerated-vehicle maintenance, replacement of last-mile cold boxes and carriers with freeze-preventive models, and extension of cold chain points.

## 1. Introduction

All vaccines are temperature-sensitive: all degrade with heat, and some, particularly those containing aluminum-based adjuvants, can be irreversibly damaged by freezing [1,2]. The requirement to store and transport vaccines at 2–8 °C throughout the supply chain is a well-established World Health Organization (WHO) standard [3]. When this range is breached, vaccines may lose potency without any visible change in appearance, meaning that children can be vaccinated with an ineffective product, with consequences ranging from inadequate protection to disease outbreaks and wasted public health investment [4,5]. Temperature excursions are not confined to low-income settings. Studies in Europe, North America, and Australasia have documented accidental exposure to damaging temperatures during both storage and transport [6,7]. These findings led WHO to recommend that all countries conduct a temperature monitoring study (TMS) at least once every five years, as part of its Effective Vaccine Management (EVM) framework, to document where in the supply chain vaccines are at risk and to provide an evidence base for corrective action [8].

Route-level TMS data from diverse settings reinforce the value of this approach. Studies in India and Lao PDR have documented substantial sub-zero and heat exposure at peripheral storage and transit points, including a shake-test failure rate of two-thirds among returned DPT vials in India [9,10]. Importantly, aggregate compliance metrics can mask such route-level vulnerabilities, a pattern also seen in Mexico and Ukraine and discussed further below [11,12]. Countries with difficult terrain, unreliable electricity, and long last-mile distances face particularly acute cold-chain challenges [13]. Nepal exemplifies these challenges, compounded by large seasonal temperature swings across a multi-tier supply chain. A field evaluation of freeze-preventive vaccine carriers in eastern Nepal recorded ambient temperatures below WHO’s minimum rated testing threshold at one site, confirming that route-level freeze risk is operationally real [14]. Yet current evidence for Nepal lacks the end-to-end, seasonally representative scope needed to identify specific storage and transport points where breaches occur across the full national chain.

Nepal’s first national TMS, conducted in 2015–2016 and reported in 2018, offers a partial picture of this vulnerability. Following the WHO study protocol for temperature monitoring in the vaccine cold chain, it tracked a single freeze-sensitive proxy vaccine across four districts and eight vaccine sub-stores [15]. Two of the four districts recorded sub-zero temperatures at the sub-store level, reaching −5.6 °C in one facility and remaining below 2 °C for up to 444 h. The other two districts, though they did not reach sub-zero, recorded temperatures above 8 °C at multiple levels. This is a heat-excursion risk for OPV. The study attributed part of this to vaccine sub-stores relying on domestic refrigerators rather than WHO-prequalified equipment. However, the study’s scope was limited to four districts and one proxy vaccine, and its fieldwork was disrupted by the 2015 earthquake and the 2015-2016 measles–rubella campaign, leaving the seasonally representative, end-to-end national picture incomplete.

Following recommendations from Nepal’s 2021 EVM assessment, a second, more robust and expanded national TMS was commissioned in 2025 with winter and summer phases across all seven provinces. This creates a timely opportunity to identify actionable cold-chain risks under real operating conditions. Nepal’s environmental gradient is extreme: winter sub-zero conditions in mountainous districts contrast sharply with summer temperatures in the Terai plains routinely exceeding 40 °C. Its immunization supply chain runs from a single Central Vaccine Store (CVS) in Kathmandu through seven Provincial Vaccine Stores (PVS), district-level stores, and in some cases, an additional cold chain point, before reaching service delivery points (SDPs) at health posts. Along this chain, vaccines move by refrigerated truck, then by cold box carried over roads that range from paved highways to seasonal mountain tracks, through elevation changes of several thousand meters. Combining end-to-end monitoring with route-level analysis across these contrasting seasons can better localize where excursions occur and support targeted system strengthening, with implications for comparable settings internationally. If route-level vulnerabilities remain hidden, damaging heat or freezing exposure may continue unnoticed, risking reduced vaccine potency, inadequate protection, and avoidable programmatic waste. This study therefore aimed to assess vaccine storage and transport conditions end-to-end across Nepal in winter and summer, identify where temperature breaches occur along the supply chain, and provide route-level evidence to target immunization supply chain strengthening. We addressed three specific objectives: (i) quantify time-in-range (2–8 °C) for all seven supply-chain routes; (ii) characterize the location, duration, and magnitude of sub −2 °C and >8 °C excursions; (iii) compare performance between winter and summer.

## 2. Materials and Methods

### 2.1. Study Design

A national temperature monitoring study was conducted in two sequential seasonal rounds: a winter round (Study 1; January to March 2025) and a summer round (Study 2; April to July 2025). The two-season design was used to capture both freeze and heat risks under the climatic extremes experienced by Nepal’s vaccine supply chain. Both rounds followed the WHO TMS methodology [3,8], adapted to local conditions.

### 2.2. Study Routes and Site Selection

The CVS and all seven PVS were included in both studies, giving seven monitoring routes, one per province. Within each province, one District Vaccine Store (DVS) was selected on the basis of the province’s predominant terrain and proximity to the central store [16]: Mountain (Taplejung, Route 1; Manang, Route 4; Humla, Route 6), Hill (Gulmi, Route 5; Bhaktapur, Route 3), and Terai (Bara, Route 2; Kanchanpur, Route 7) (Figure 1). District selection combined terrain representation with deliberate inclusion of two remote districts (Taplejung and Humla, farthest from the central store) and one highly accessible district (Bhaktapur, nearest to the central store).

Within each selected district, one SDP was then chosen by a lottery method from a list, provided by UNICEF, of all SDPs providing vaccination services at the lowest supply chain level. Because this step was randomized rather than purposively targeted, two of the routes drawn (Routes 1 and 7) were found to pass through an existing cold chain point between the DVS and the SDP, an additional supply chain level, so they were retained once identified. In total, 24 facilities across five supply chain levels were covered (Table 1). The site-selection strategy was designed to provide geographic and operational coverage across all seven provinces and to capture variation in terrain and accessibility. It was not intended to constitute a statistically representative probability sample of all vaccine storage and service delivery facilities in Nepal.

### 2.3. Vaccines Monitored

Two vaccines under the National Immunization Program were selected based on their contrasting thermal sensitivities: Pentavalent vaccine, which is freeze-sensitive; and oral polio vaccine (OPV), which is heat-sensitive. This pairing allowed the study to assess risks at both ends of the temperature spectrum.

The Pentavalent vaccine used was a liquid, whole-cell pertussis-based diphtheria-tetanus-pertussis-hepatitis B-Haemophilus Influenza type b (Hib) formulation, adsorbed on aluminum phosphate adjuvant (WHO-prequalified), supplied in 10-dose vials in the country. The vaccine requires storage and transport at 2–8 °C and must not be frozen. The OPV used was a live attenuated, bivalent (types 1 and 3) oral polio vaccine, WHO-prequalified, supplied in 10-dose vials with a vaccine vial monitor type 2, the category most sensitive to cumulative heat exposure. The vaccine remains potent when stored at −20 °C until expiry or for up to six months between 2–8 °C.

### 2.4. Temperature Monitoring Equipment

Each shipment was accompanied by two WHO-prequalified electronic data loggers, both preset to record temperature at 30-min intervals: a LogTag UTRID-16, the standard logger specified for temperature monitoring studies under WHO guidelines and used as the primary device, and a Vac-TL-01, used as a backup logger. Data were retrieved from the LogTag UTRID-16 for all routes with available data; the Vac-TL-01 served only to provide a temperature record where the primary logger failed. Vaccine vial monitors (VVMs) on the monitored vials, which in use throughout the immunization supply chain, provided an independent cumulative indicator of heat exposure at the point of vaccination.

### 2.5. Data Collection Process

A set of standardized forms: nine in Study 1 (winter) and ten in Study 2 (summer) was generated from a Nepal-specific Excel-based TMS tool developed for this study. Forms captured the time, ambient temperature, GPS coordinates, and storage and transit equipment details at each arrival and dispatch. Field staff photographed completed forms and shared them through a dedicated means of communication for real-time tracking. No patient or beneficiary data was collected or shared at any stage; the forms captured facility-level operational data only, including vaccine batch numbers, storage temperatures, facility GPS coordinates, and staff contact details recorded for data verification purposes. On completion of each route, data loggers were returned to the CVS, and recorded data was downloaded directly into the TMS tool, minimizing transcription error. Among the seven monitored routes, winter data from the Karnali route were unavailable because the logger data from both the primary and backup loggers were corrupted due to an undiagnosed error and could not be recovered, despite attempts to retrieve the data, including consultation with the logger manufacturer.

### 2.6. Training

Fourteen provincial cold chain inspectors (two per province) received a half-day training in study methodology, form completion, and sensor management. These inspectors then served as master trainers for district and SDP staff. The training was designed both for data quality and as a capacity-building measure: provincial teams were to be capable of conducting future TMS independently.

### 2.7. Data Analysis

Data were processed using a customized, Excel-based TMS tool (Version 1.0), developed specifically for this study. The TMS tool generated time-temperature profiles for each route and segment. The route was the primary unit of analysis for overall cold-chain performance. For each route, the percentage of monitored time within the recommended 2–8 °C range and MKT were calculated. Route-segment analyses were additionally conducted to characterize the location, duration and magnitude of temperature excursions below 2 °C and above 8 °C along the supply chain. Cumulative monitoring time was used to quantify the duration of temperature exposure within and outside the recommended range. MKT was calculated using the Arrhenius equation, providing a single-value indicator of cumulative thermal exposure that is weighted toward higher temperatures [17]. The following equation was used to calculate the MKT value:

$$T_{K}=\frac{\frac{∆H}{R}}{-ln\;\left(\frac{t_{1}e\;\left(\frac{-∆H}{{RT}_{1}}\right)+\;t_{2}e\left(\frac{-∆H}{{RT}_{2}}\right)+\cdots +\;t_{n}e\left(\frac{-∆H}{{RT}_{n}}\right)}{t_{1}+\;t_{2}\;+\cdots +\;t_{n}}\right)}$$

where:

$T_{K}\;is\;the\;mean\;kinectic\;temperature\;in\;kelvins$

$∆H\;is\;the\;activation\;energy\;(typically\;within\;42–125\;kJ\;{mol}^{-1}\;for\;solids\;or\;liquids\;=\;83.14472)$

R is the gas constant = 0.0083144

$T_{1}\;to\;T_{n}\;are\;the\;temperatures\;at\;each\;of\;the\;sampel\;points\;in\;kelvins$

$t_{1}\;to\;t_{n}\;are\;time\;intervals\;at\;each\;of\;the\;sampel\;points$

Given the small number of monitored routes (six in winter and seven in summer) and the study’s operational sampling approach, route-level differences were summarized descriptively using route-specific values, means, medians, and ranges. No inferential statistical comparisons between routes or seasons were performed.

Route-level MKT was summarized in the same descriptive way (mean, median, and 95% CI), using standard t-distribution methods, since MKT is a continuous, unbounded measure and does not require the logit transformation applied to the bounded % safe outcome. The duration and temperature extremes of all excursions below 2 °C and above 8 °C were recorded for each route and segment.

### 2.8. Ethical Approval

The study protocol was approved on 19 June 2025 by the Nepal Health Research Council (NHRC), Ref. No.: 2817.

## 3. Results

### 3.1. Study Duration and Completeness

Study 1 ran from 1 January to 31 March 2025. Six of seven routes were completed. Total monitoring time for the six completed routes was 7787 h (324 days and 11 h). Monitoring duration per route ranged from 50 days 23 h (Sudurpashchim) to 58 days 20 h and 30 min (Madhesh). Study 2 ran from 9 April to 4 July 2025, with all seven routes completed. Total monitoring time was 10,400 h (433 days and 8 h). Monitoring duration per route ranged from 43 days 23 h (Sudurpashchim) to 85 days 22 h (Karnali), reflecting differences in transit distance and storage time at intermediate facilities.

### 3.2. Overall Cold Chain Performance

Table 2 summarizes the aggregate findings. In Study 1 (winter), vaccines were within the safe range for 98.1% of total monitored time; in Study 2 (summer), for 97.3% of total monitored time. Across the full monitoring period, freeze exposure was more common than heat exposure (272.5 h vs. 155.0 h). This asymmetry was concentrated in winter, when heat excursions were rare (18.0 h, 0.2% of monitored time) against extensive freeze exposure (132.0 h, 1.7%); in summer, the two were comparable in magnitude (1.4% vs. 1.3% of monitored time). MKT remained within 2–8 °C for every route in both seasons: 5.5–6.1 °C in winter, 2.6–4.6 °C in summer. With six (Study 1, winter) and seven (Study 2, summer) routes, the limited number of routes provided insufficient power to detect modest between-route differences, and comparisons were therefore presented descriptively rather than through formal statistical tests. In winter, route-level compliance ranged from 94.5% (Koshi) to 99.6% (Bagmati) (mean 98.1%, median 98.3%, 95% CI 96.3–99.6%). In summer, route-level compliance ranged from 95.4% (Koshi) to 99.7% (Bagmati) (mean 97.4%, median 97.5%, 95% CI 95.4–99.1%).

### 3.3. Temperature Excursions Below 2 °C (Freeze Risk)

Vaccines were exposed to sub −2 °C temperatures for a cumulative 132.0 h in Study 1, winter (1.7% of monitored time) and 140.5 h in Study 2, summer (1.4%) (Table 2). The lowest recorded temperature was −12.1 °C, recorded during walk-in cold room storage at the provincial vaccine store on the Bagmati route in winter (Table S1).

Segment-level analysis showed that sub −2 °C exposure was concentrated at the cold chain point and service delivery point levels, though not exclusively so (Tables S1 and S2, Figure 2). Storage at these two levels accounted for 40.5% of winter’s and 56.2% of summer’s freeze hours, and transit into the cold chain point or service delivery point added a further 11.7% and 8.9%, for a combined 52.3% in winter and 65.1% in summer. The single largest contributor was cold-box storage at the cold chain point on the Koshi route in winter: 34.5 of the winter’s 132 total freeze hours (26.1%), representing 90.8% of that segment’s own monitored time. Similarly concentrated freeze exposure occurred at the Gandaki DVS in winter (13.0 of that segment’s 14.0 monitored hours, 92.9%), the Karnali HP in summer (21.5 of 42.0 h, 51.2%), and the Sudurpashchim hospital in winter (16.5 of 27.0 h, 61.1%), indicating that near-continuous freeze exposure at a single facility was not unique to Koshi. The remaining freeze exposure was distributed across walk-in cold room and refrigerator storage at the provincial and district stores and the outreach leg to vaccination sessions. Freeze exposure was also unevenly distributed across routes; this is examined by terrain in Section 3.6.

### 3.4. Temperature Excursions Above 8 °C (Heat Risk)

Heat excursions totaled 18.0 h in Study 1, winter (0.2%) and 137.0 h in Study 2, summer (1.3%) (Table 2). Summer heat exposure was roughly 7.6 times higher than in winter. The maximum recorded temperature was 31.5 °C, recorded during walk-in cold room storage at the CVS on the Koshi route in summer (Table S2).

Segment-level analysis showed that heat exposure was concentrated at two points: storage at the central and provincial vaccine stores, and transit between them (Figure 3). Combined, these two categories accounted for 63.9% of winter heat hours and 73.0% of summer heat hours (Tables S1 and S2, Figure 3). The single largest contributor was the transit leg from the central store to the provincial store on the Karnali route in summer: 22.0 of that season’s 137.0 total heat hours (16.1%), representing 71.0% of that segment’s own monitored time. One facility recorded both risks at once in summer, an exception examined in Section 3.6.

A separate case, specific to winter, is worth noting on its own. On Route 2 (Madhesh), the journey from the PVS to the DVS took 2 days, 6 h, and 30 min, against an expected transit time of approximately 5 h; the same leg took 5 h and 30 min when monitored in summer under normal conditions. Temperatures during the delay itself stayed within range, but direct review of the time-temperature record for this segment showed temperatures fluctuated above and below 8 °C at the start of the leg and again over the final several hours as the shipment neared DVS, cumulatively exceeding the threshold for 2.5 h, 13.9% of winter’s total heat exposure on its own (Figure 4).

### 3.5. VVM Status at the Point of Vaccination

VVM readings at the point of vaccination were all at a usable stage in both studies. However, slightly faded VVM readings for OPV were observed at the final service delivery point on two of seven routes (Koshi, Lumbini), recurring in both winter and summer at each.

### 3.6. Route-Level Performance

Table 3 presents route-level results for both studies, with each route’s own monitoring denominator so that excursion hours can be read as rates. Bagmati (Route 3) had the highest safe-range compliance in both seasons (99.6% winter, 99.7% summer). Koshi (Route 1) had the lowest in both seasons (94.5% winter, 95.4% summer), driven largely by freeze exposure in cold-boxes used for storage at facilities beyond the district store, one of the most remote mountainous districts in the country: 34.5 h at PHCC in winter, 51.1% of that route’s winter freeze exposure, and 40.5 h at HP in summer, 58.3% of its summer total (Tables S1 and S2). At the same health post in summer, freeze and heat exposure overlapped: 40.5 of its 71.5 monitored hours fell below 2 °C, and a further 9.0 h rose above 8 °C, leaving only 22.0 h (30.8%) within the safe range, the clearest example in the dataset of the winter-freeze, summer-heat pattern not holding at every facility.

Grouping routes by terrain showed that the two risks separated by season and geography (Table 3). In winter, Mountain routes spent 3.3% of their monitored time below 2 °C (87.0 h of 2612.5), against 1.4% on Terai routes (38.0 of 2635.5) and 0.3% on Hill routes (7.0 of 2539.0). Heat exposure showed the opposite pattern in summer, when Terai routes spent 1.9% of monitored time above 8 °C (58.0 h of 3106.5), the highest heat rate recorded for any terrain in either season, compared with 1.1% on Mountain routes (56.5 h of 5088.5) and 1.0% on Hill routes (22.5 of 2205.0). Freeze risk on Mountain routes did not resolve with the season, remaining at 2.7% of monitored time in summer (137.5 h of 5088.5), while Terai freeze exposure fell from 38.0 h (1.4%) in winter to 1.0 h (under 0.1%) in summer. Mountain routes therefore carried the highest freeze exposure in both seasons, whereas heat exposure was seasonal and concentrated on Terai routes. With two to three routes in each terrain group, and Karnali contributing to the summer Mountain group only, these comparisons were descriptive.

## 4. Discussion

The principal finding of this study is that Nepal’s vaccine cold chain maintained safe conditions for more than 97% of monitored time in both winter and summer, with MKT values consistently within the recommended 2–8 °C range and no assessed OPV VVM having reached its discard point. This is a notable result for a country whose supply chain crosses some of the most difficult terrain in the world, and it reflects the investment in cold chain infrastructure made over the past decade, including walk-in cold rooms at central and provincial levels and solar refrigerators at many district stores [18]. WHO does not specify a single global cutoff for “percentage time in range”, defining excursions instead by temperature and duration thresholds [8,16]. Differences in monitored duration, route selection and reporting metrics mean these figures position Nepal’s result rather than rank it.

International temperature monitoring studies show a similar pattern. In Mexico, storage and transport remained within range for 92% of monitored time, but temperature variation widened progressively toward service points [11]. Ukraine recorded near-perfect compliance at national and subnational stores, with transport compliance falling to as low as 54.8% at primary healthcare centers [12]. India documented sub-zero exposure for 10.5% of peripheral storage time and approximately 18% of transit time [9]. In Lao PDR, heat exposure above 8 °C occurred mostly during transit from the capital to the province, while the less frequent freeze exposure was highest at district-level storage [10]. Read together, these four studies place compliance highest at central levels and lowest toward the last mile, the same structural pattern this study’s segment-level findings show for Nepal.

That said, the aggregate numbers conceal risks that deserve close attention. Operational vulnerabilities that could compromise vaccine potency persist beneath reassuring headline metrics, examined in turn below:

### 4.1. Freeze Risk on the Last Mile

Freeze exposure during last-mile cold-box transport is easy to underestimate because the study’s primary thermal indicator, MKT, is not built to detect it. MKT is based on the Arrhenius equation, which is weighted toward higher temperatures and is relatively insensitive to brief or moderate drops below 0 °C [17]. As a result, MKT can remain within the safe range even when individual episodes of sub-zero exposure would have physically damaged freeze-sensitive vaccines. However, this study did not include shake testing, a validated field method for detecting freeze damage in aluminum-adjuvanted vaccines [19,20]; without it, the number of freeze-damaged vaccine vials could not be determined. Future rounds of the TMS in Nepal should incorporate shake testing as standard procedure at the point of distribution for any route that records sub-zero temperatures, to confirm vaccine integrity directly rather than inferring risk from temperature data alone.

The exposure data show why this matters. Vaccines were below 2 °C for a cumulative 272.5 h across both studies, with temperatures reaching as low as −12.1 °C (Bagmati, winter) and −6.5 °C (Gandaki, summer). For the Pentavalent vaccine, which can be irreversibly damaged by a single freeze-thaw cycle through adjuvant clumping that reduces immunological properties [21], these are not marginal readings. Hanson et al. reviewed 35 years of evidence and concluded that freezing in the vaccine cold chain is an ongoing, widespread, and under-recognized issue [6]. Studies in India [9], Cameroon [22], and Mexico [11] have all identified cold-box transport as the segment with the highest freeze risk, and Nepal’s segment-level findings show this freeze exposure concentrated in cold-box and vaccine-carrier storage and transit toward the last mile, consistent with inconsistent field-level practice during the final stages of vaccine movement; frozen or inadequately conditioned ice packs placed in direct contact with vaccine vials are the most likely explanation of the observed sub-zero exposures as stated by earlier studies [7,9]. This, however, does not explain the remaining freeze exposures recorded during walk-in cold room and refrigerator storage, where ice packs play no part. This points to a separate, and at least partly infrastructural, gap that this study’s data cannot further characterize.

Nepal-specific field evaluations of freeze-preventive vaccine carriers [14] and freeze-preventive cold boxes [23] have demonstrated that equipment designed to buffer vaccines from frozen packs can reduce last-mile freezing risk. A quick fix can be icepack conditioning training [24] or replacing frozen-water packs with phase-change material packs cooled to +5 °C that has been shown to reduce transport freezing risk from 13.8% to 1.7% in Tunisia [25]. Other solutions may include deployment of freeze-preventive cold boxes and vaccine carriers or extension of cold chain points focusing on the SDPs. These solutions are operationally feasible and may require less capital investment.

### 4.2. Seasonal Heat Risk

The seasonal shift from freeze risk (winter) to heat risk (summer) at the same facilities pointed to weaknesses that are both operational and infrastructural. Heat excursions above 8 °C were roughly 7.6 times as frequent in summer as in winter. The two main sources were uncontrolled ambient exposure during loading and unloading, where no temperature-regulated loading area existed at the central and provincial stores, and possibly impaired temperature control in refrigerated vehicles on the transit leg; equipment condition was not recorded in this study, so the vehicle explanation cannot be confirmed. A transit delay was recorded on the Madhesh, PVS to DVS leg in winter: 2 days, 6 h, and 30 min, compared to 5 h 30 min for the same leg during the summer study; this illustrates weaknesses in shipment planning and supervision. Cold chain assessments in other low- and middle-income settings, including Ukraine, have repeatedly identified loading, unloading, and transport as vulnerable points [12,13]. These are both correctable through procedural changes and equipment maintenance, without new capital investment [26,27].

### 4.3. VVM as an Early-Warning Signal

The VVM findings deserve a cautionary interpretation. The absence of unusable VVM readings might be taken as evidence that no vaccine was wasted. But the appearance of faded VVM for OPV across Koshi and Lumbini routes in both studies indicated that vaccines arrived at the point of vaccination with reduced thermal tolerance. One additional adverse event, a power outage, a longer transit, or an equipment failure could result in large-scale wastage [28,29].

### 4.4. Synthesis: From Aggregate Metrics to Operational Risk

Taken together, these findings highlight the importance of distinguishing overall cold-chain capacity from operational performance at specific points in the vaccine supply chain. While aggregate cold-chain performance was high, residual risks were concentrated at operational interfaces, particularly last-mile transport, vaccine handling during loading and unloading, transit management, and equipment maintenance. This mirrors the pattern reported elsewhere [11,12]. Addressing these vulnerabilities may therefore require a combination of appropriate equipment and strengthened operational practices, including updated standard operating procedures, training of cold-chain personnel, supportive supervision, preventive equipment maintenance, and routine use of temperature-monitoring data to guide corrective action. Future rounds could build this into an explicit loop, risk-differentiated monitoring informed by the terrain and seasonal pattern above, feeding into early warning and intervention, verified through the shake testing and VVM review discussed in this section, and followed by corrective and preventive action, rather than treating each TMS as a standalone assessment.

### 4.5. Strengths and Limitations

Our study has several strengths. It included all seven provinces, providing broad geographic coverage across Nepal’s highly challenging terrain. The two-season design captured both winter freeze risk and summer heat risk. The route-level approach allowed identification of specific points of failure that aggregate national metrics would obscure. The custom Excel-based monitoring tool and train-the-trainer model, in which provincial cold chain inspectors trained centrally went on to train district and facility-level staff, enhance feasibility and transferability to other programs. Importantly, the main risks identified are operationally correctable without requiring major new capital investment.

However, several limitations should be considered too when interpreting these results. The sample (24 facilities across 7 provinces) is a snapshot, not a census; performance at non-sampled facilities may differ. Selection of a single DVS per province to represent the area’s predominant terrain, while deliberately including two remote districts (Taplejung and Humla) and one highly accessible district (Bhaktapur), further limits generalizability to non-selected districts within each province, particularly those with intermediate levels of remoteness. Statistical comparisons across routes were limited to descriptive summaries rather than inferential testing: the 30 min logger readings within a route are temporally correlated rather than independent, and with only seven monitored routes, route-level predictors are collinear with route identity itself, so a model built on this number of clusters would not be reliably estimable. Transit delay was examined descriptively but could not be analyzed systematically: the study tool’s reference travel times are planning values rather than measured baselines, logged transit segments include holding time at either end, and comparing the same leg across seasons confounds duration with season. Data were lost for one route in Study 1 (Karnali, winter), limiting comparability of seasonal results for the province.

The 30-min recording interval means that situations at transit stops shorter than 30 min may not be captured. Ambient temperature was recorded only twice per facility (at arrival and dispatch), not continuously, and equipment condition, including ice-pack conditioning state and the working status of refrigerated vehicles, was not captured by the data collection forms at all. The study may be affected by the Hawthorne effect: field staff knew they were being observed, and may have performed better than under routine, unmonitored conditions. The study also did not measure vaccine wastage rates or immunization coverage outcomes alongside temperature data; the relationship between temperature compliance and actual program outcomes therefore remains inferential rather than directly demonstrated. The reported results should therefore be interpreted as one operational scenario rather than a description of typical practice.

Nepal’s first national TMS (2015–2016) recorded sub-zero exposure of up to 444 h at a single sub-store relying on domestic refrigeration, and heat excursions at facilities using similar equipment [15]. A decade of investment in walk-in cold rooms and solar refrigeration appears to have reduced the severity of exposure at any single facility, though the present study’s broader sampling and two-season design mean the two studies are not directly comparable, and freeze and heat risks both remain present across the current sample. This suggests infrastructure investment has helped without resolving the operational and last-mile gaps identified in this study.

## 5. Conclusions

Nepal’s vaccine cold chain maintained recommended temperatures for more than 97% of monitored time across both seasons, but route-level monitoring identified important residual risks that were not apparent from aggregate measures. Because MKT is heat-weighted and no shake testing was performed, this aggregate figure cannot confirm that the Pentavalent vaccine on routes with sub-zero exposure retained potency. Freeze exposure was concentrated in last-mile transport and storage, while heat exposure occurred predominantly during summer transport and vaccine handling. These findings underscore the importance of complementing aggregate cold-chain indicators with route-level monitoring and strengthening operational practices, including temperature-regulated loading areas in vaccine stores to reduce the risk of extreme ambient exposure, capacity building of health personnel, ice-pack conditioning, transport procedures, equipment maintenance, use of freeze-preventive cold boxes and carriers, extension of cold chain points, and continuous temperature monitoring. Integrating periodic temperature monitoring and corrective action into routine immunization program management could support continuous improvement of vaccine supply-chain performance. A future round of monitoring designed to support formal inferential modeling would need a substantially larger number of routes, together with consistently recorded per-visit ambient temperature, equipment condition, and standardized expected transit times for each leg. These findings come from a purposively selected sample of routes and facilities rather than a nationally representative one, and should be read as illustrating the vulnerabilities such a supply chain can face rather than a national census of performance.

## Figures and Tables

**Figure 1:**
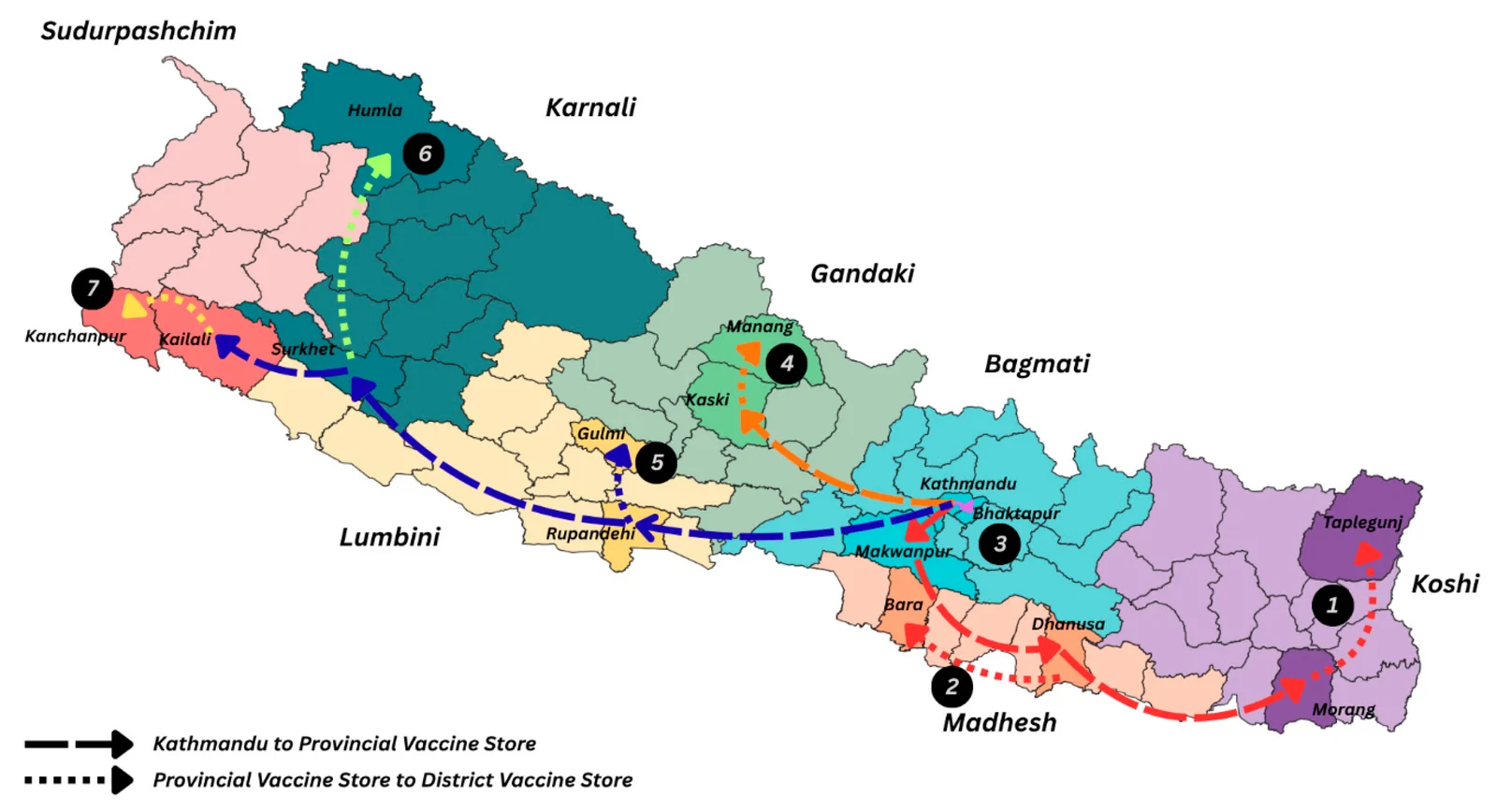
Vaccine transportation routes from CVS to seven provinces. The arrows have meaning that the sample moved toward a specific direction.

**Figure 2:**
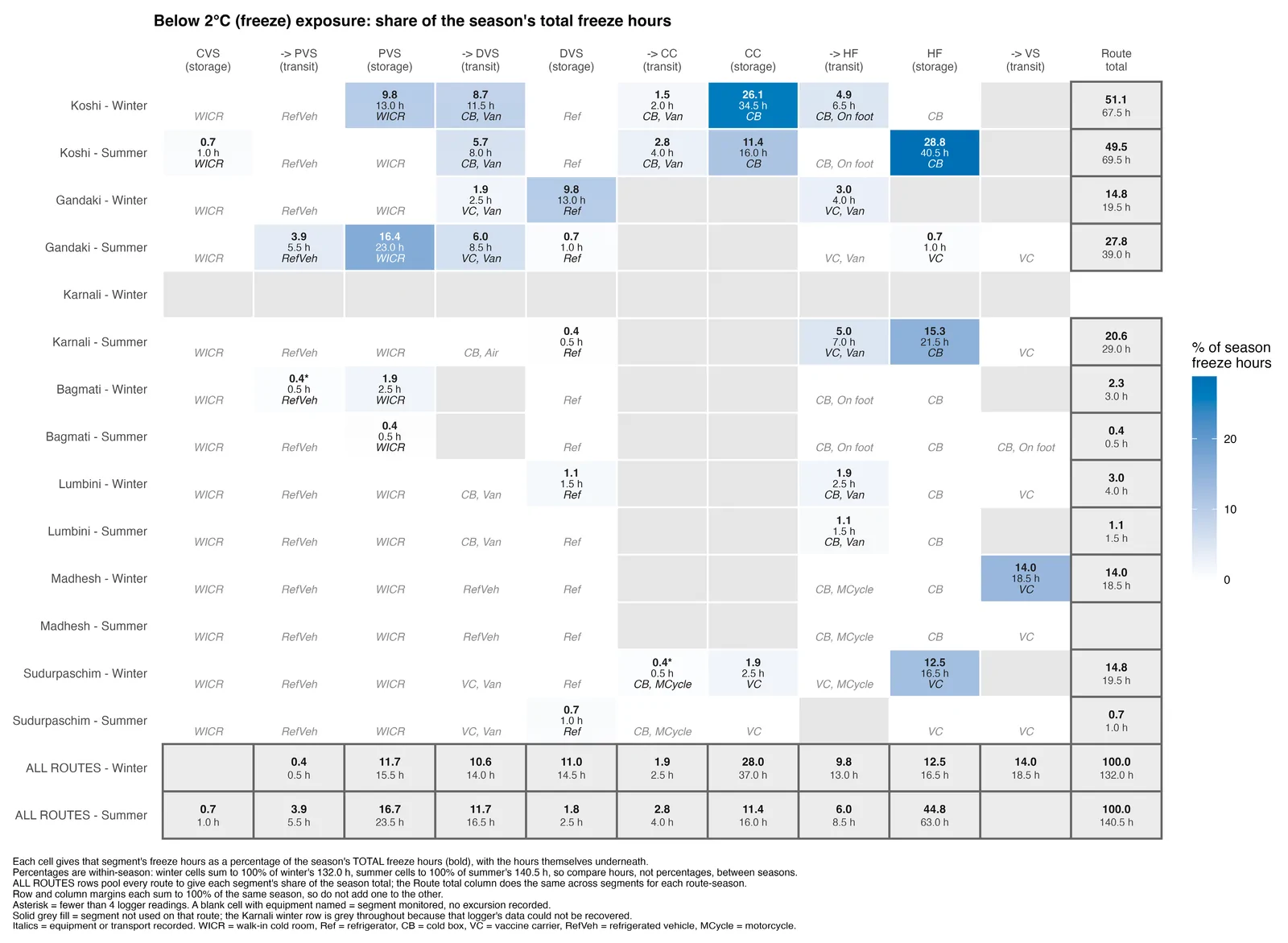
Freeze (<2 °C) exposure as each segment’s share of the season’s total freeze hours.

**Figure 3:**
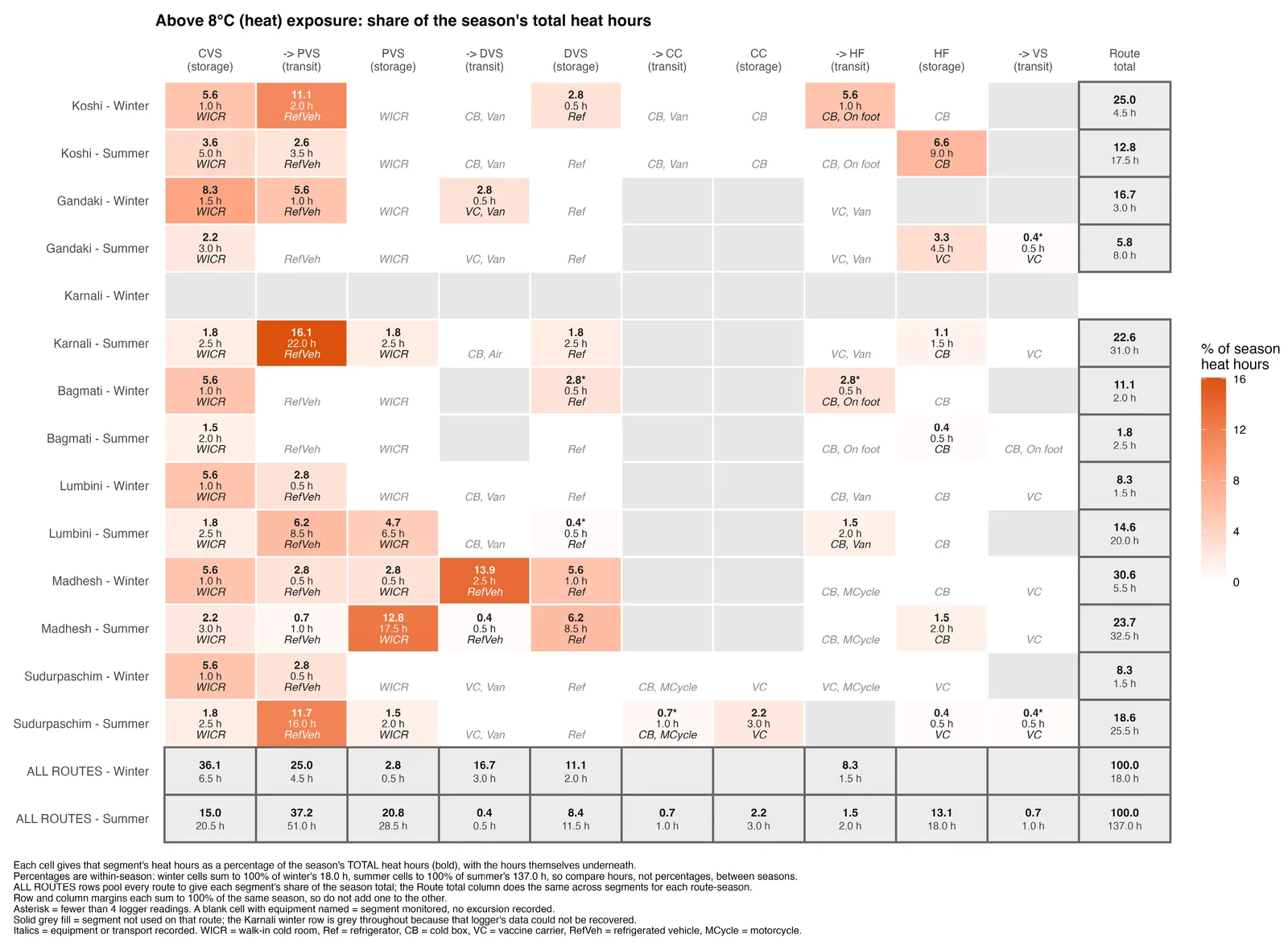
Heat (>8 °C) exposure as each segment’s share of the season’s total heat hours.

**Figure 4:**
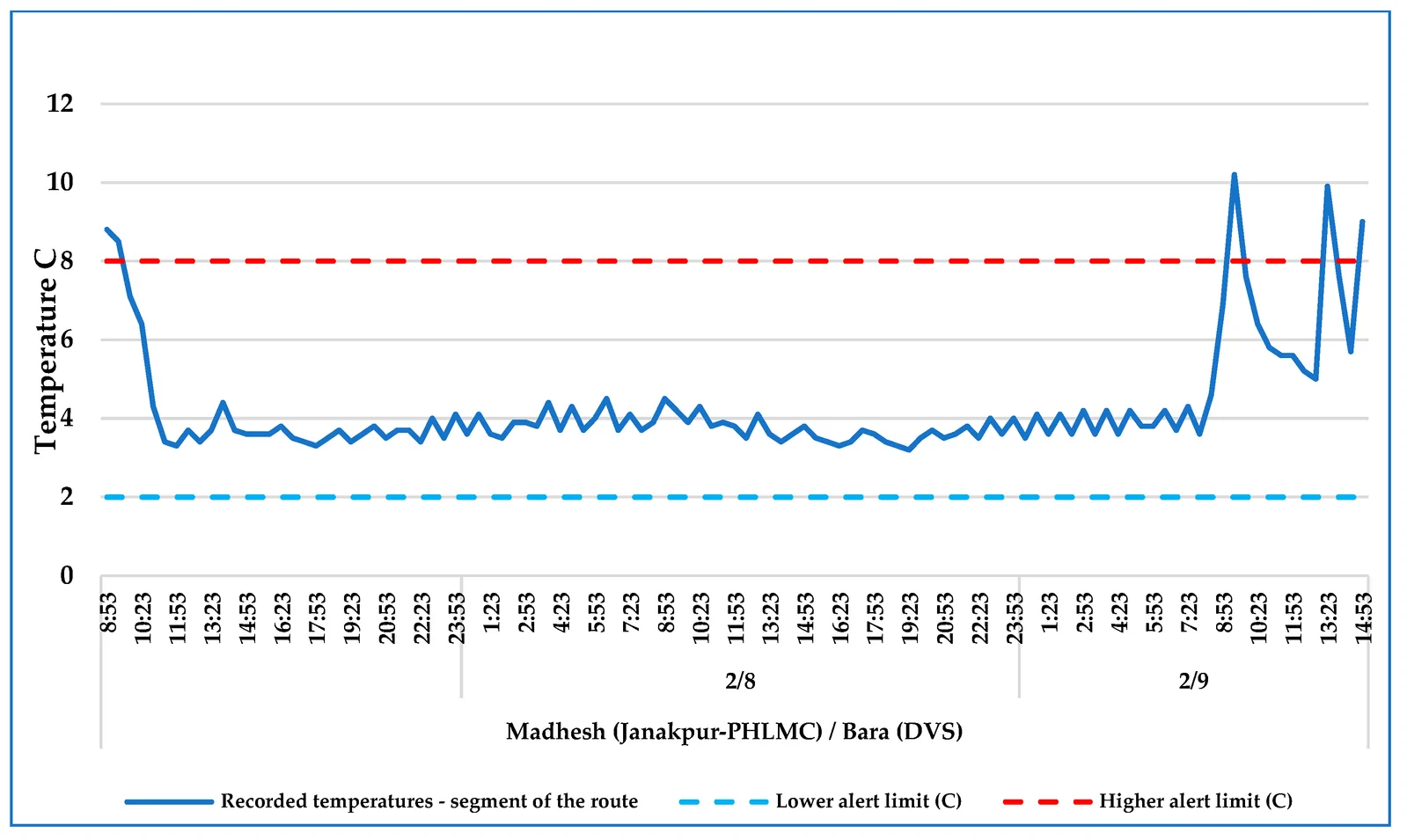
Recorded temperature during transit from PVS to DVS, Route 2 (Madhesh), Study 1 (winter).

**Table 1:** Study sites by province, supply chain level, and terrain type.

Route	Province	PVS (PHLMC)	DVS (District)	Terrain	CC Point	SDP
1	Koshi	Biratnagar	Taplejung	Mountain	Dungeshangu PHC	Phakamba HP
2	Madhesh	Janakpur	Bara	Terai	–	Kabaigoth HP
3	Bagmati	Bhaktapur	Bhaktapur	Hill	–	Duwakot HP
4	Gandaki	Pokhara	Manang	Mountain	–	Dharapani HP
5	Lumbini	Butwal	Gulmi	Hill	–	Dhurkot HP
6	Karnali	Surkhet	Humla	Mountain	–	Saya HP
7	Sudurpashchim	Dhangadhi	Kanchanpur	Terai	Dudhara Chandani Municipality	Dudhara Chandani Municipality Hospital

PVS = Provincial Vaccine Store; PHLMC = Provincial Health Logistics Management Center; DVS = District Vaccine Store; CC = cold chain; SDP = service delivery point; HP = health post.

**Table 2:** Aggregate cold chain performance in Study 1 (winter) and Study 2 (summer).

Studies	Total Duration (hours)	Time in Safe Range, hours (%)	Time <2 °C, hours (%)	Time >8 °C, hours (%)	MKT Range (°C)
Study 1 (Winter)	7787.0	7637.0 (98.1)	132.0 (1.7)	18.0 (0.2)	5.5–6.1
Study 2 (Summer)	10,400.0	10,122.5 (97.3)	140.5 (1.4)	137.0 (1.3)	2.6–4.6

MKT = mean kinetic temperature. Study 1 includes six routes (Karnali winter data not available due to recording failure); Study 2 includes all seven routes.

**Table 3:** Route-level temperature performance by province and season.

		Winter					Summer				
Province (Route)	Terrain	Monitored (h)	% Safe	MKT (°C)	<2 °C, h (%)	>8 °C, h (%)	Monitored (h)	% Safe	MKT (°C)	<2 °C, h (%)	>8 °C, h (%)
Koshi (1)	Mountain	1319.0	94.5	5.6	67.5 (5.1)	4.5 (0.3)	1898.0	95.4	4.3	69.5 (3.7)	17.5 (0.9)
Gandaki (4)	Mountain	1293.5	98.3	6.1	19.5 (1.5)	3.0 (0.2)	1128.5	95.8	2.7	39.0 (3.5)	8.0 (0.7)
Karnali (6)	Mountain	—*	—*	—*	—*	—*	2062.0	97.1	4.6	29.0 (1.4)	31.0 (1.5)
Bagmati (3)	Hill	1294.5	99.6	5.6	3.0 (0.2)	2.0 (0.2)	1079.0	99.7	2.6	0.5 (0.1)	2.5 (0.2)
Lumbini (5)	Hill	1244.5	99.6	5.6	4.0 (0.3)	1.5 (0.1)	1126.0	98.1	2.8	1.5 (0.1)	20.0 (1.8)
Madhesh (2)	Terai	1412.5	98.3	5.5	18.5 (1.3)	5.5 (0.4)	2051.5	98.4	4.4	0.0 (0.0)	32.5 (1.6)
Sudurpashchim (7)	Terai	1223.0	98.3	5.8	19.5 (1.6)	1.5 (0.1)	1055.0	97.5	2.6	1.0 (0.1)	25.5 (2.4)
All routes		**7787.0**	98.1		**132.0 (1.7)**	**18.0 (0.2)**	**10,400.0**	97.3		**140.5 (1.4)**	**137.0 (1.3)**
Mean (95% CI) †			98.1 (96.3–99.6)	5.7 (5.5–5.9)				97.4 (95.4–99.1)	3.4 (2.6–4.3)		
Median			98.3	5.6				97.5	2.8		

Hours <2 °C and >8 °C are cumulative for the entire route. —* Karnali winter data not available due to recording failure. † Winter summary based on *n* = 6 routes (Karnali excluded); summer based on *n* = 7 routes. A 95% CI was calculated on the logit scale and back-transformed; MKT 95% CI uses standard t-distribution methods, as MKT is a continuous, unbounded measure. Segment-level temperature monitoring and equipment data are reported in Supplementary Tables S1 (winter) and S2 (summer).

## Data Availability

The datasets presented in this article are not readily available because they contain facility-level information collected under conditions of confidentiality. Requests to access the datasets should be directed to the corresponding author.

## Supplementary Materials

The following supporting information can be downloaded at: https://www.mdpi.com/article/10.3390/vaccines14100837/s1, Table S1: Study1-Segment-level temperature monitoring and equipment data by route, winter; Table S2: Study2-Segment-level temperature monitoring and equipment data by route, summer.
